# Behavioral correlates of subjective cognitive decline in the Canadian Longitudinal Study on Aging (CLSA)

**DOI:** 10.1093/geroni/igab046.2763

**Published:** 2021-12-17

**Authors:** Shawna Hopper, Nicole Hammond, Arne Stinchcombe

**Affiliations:** 1 Brock University, St Catharines, Ontario, Canada; 2 University of Ottawa, Ottawa, Ontario, Canada; 3 Brock University, St. Catharines, Ontario, Canada

## Abstract

Subjective cognitive decline (SCD) is a self-reported decline in cognition among otherwise cognitively healthy older adults. It is believed that SCD may be a precursor to Alzheimer’s Disease (AD). Analyzing data from the Canadian Longitudinal Study on Aging (CLSA), a large national sample of participants aged 45-85 at baseline, we sought to identify prospective relationships between health-related behaviors and SCD. Exposures were measured at baseline and SCD was measured three years later, with the question: “Do you feel like your memory is becoming worse?”. A multivariable logistic regression model was used to estimate odds of SCD (analytic sample: n=35,680). Alcohol consumption was associated with increased odds of SCD, with regular drinkers (OR=1.13, 95% CI: 1.04, 1.22) and frequent drinkers (OR=1.17, 95% CI: 1.08, 1.27) more likely to report SCD than never drinkers. Compared to participants who never smoked, former smokers had increased odds of SCD (OR=1.13, 95% CI: 1.08, 1.18), whereas current smokers had reduced odds of SCD (OR=0.90, 95% CI: 0.83, 0.98). Participants who consumed five or more servings of fruits/ vegetables had reduced odds of SCD (OR=0.95, 95% CI: 0.91, 0.99), when compared to those who consumed <5 servings. Lastly, we did not observe any associations between walking and SCD. This study identifies relationships between various health-related behaviors and SCD in a large population-based sample of older Canadians. Identification of modifiable risk factors may help with early prevention and intervention of SCD.

